# Closed-Loop Bowel Obstruction Years After an Open Abdominal Aortic Aneurysm Repair

**DOI:** 10.7759/cureus.18586

**Published:** 2021-10-07

**Authors:** Adam J Mann, Nicholas Laconi, Robert S Smith

**Affiliations:** 1 General Surgery, Florida Atlantic University, Boca Raton, USA; 2 General Surgery, University of Florida, Gainesville, USA; 3 Acute Care Surgery, University of Florida College of Medicine, Gainesville, USA

**Keywords:** postoperative complications, small bowel obstruction, small bowel necrosis, bowel ischemia, small bowel resection, abdominal aortic aneurysms, abdominal radiology, bowel obstruction, aortic surgery, general and vascular surgery

## Abstract

A 68-year-old male has a significant past medical history of severe aortic stenosis, peripheral arterial disease, chronic kidney disease, and an abdominal aortic aneurysm treated with a bifurcated interposition aortobiiliac graft. He was admitted to the hospital for an elective one-vessel coronary artery bypass graft and placement of a bioprosthetic aortic valve. Postoperatively, he developed worsening abdominal pain, leukocytosis, and inability to tolerate nutrition by mouth. Computed tomography revealed moderately dilated loops of the small bowel with two transition points in the right lower quadrant. He was taken emergently to the operating room for an exploratory laparotomy, and a 28-cm necrotic jejunal loop was entrapped posterior to the right iliac segment of the graft. In a patient with an intra-abdominal synthetic vascular graft, a closed-loop bowel obstruction caused by entrapment by the vascular graft is exceptionally rare; however, it should be considered in the presence of bowel obstruction.

## Introduction

Closed-loop intestinal obstructions are commonly caused by adhesions, internal herniation, or volvulus and can compromise bowel perfusion and progress to intestinal necrosis and perforation [1]. Any intraperitoneal operation can create an adhesive disease that may cause bowel obstruction and require operative intervention [1]. The transabdominal, open approach to an abdominal aortic aneurysm repair is certainly no exception as this procedure involves intraperitoneal placement of a prosthetic vascular graft. Adhesive disease to intra-abdominal vascular grafts has been reported in the literature [2,3,4]. However, an extensive review of the literature revealed no reported cases of closed-loop bowel obstruction by direct entrapment of the bowel by a synthetic vascular graft. Herein, we discuss the case of a closed-loop small bowel obstruction caused by the entrapment of the jejunum posterior to an aortobiiliac Dacron graft that developed three years after abdominal aortic aneurysm repair.

## Case presentation

A 68-year-old male has a significant past medical history of severe aortic stenosis, peripheral arterial disease, chronic kidney disease, and an abdominal aortic aneurysm. He lives at home with his spouse and can independently perform routine daily activities of living. Nine years prior, he underwent an endovascular abdominal aortic aneurysm repair, which was complicated by an endoleak and required multiple graft revisions, eventual explantation of the endovascular graft, and placement of a bifurcated interposition aortobiiliac graft. The patient was admitted to the hospital for an elective one-vessel coronary artery bypass graft and placement of a bioprosthetic aortic valve. There were no intraoperative complications and the patient was extubated one day later. Four days after surgery, he developed worsening abdominal pain, leukocytosis, and inability to tolerate nutrition by mouth. Enteral nutrition was stopped, and a nasogastric tube on low intermittent suction was removed with over 1 L of bilious output daily. On postoperative day five, his abdominal pain progressed to focal peritonitis in the right lower quadrant.

Investigations

**Figure 1:**
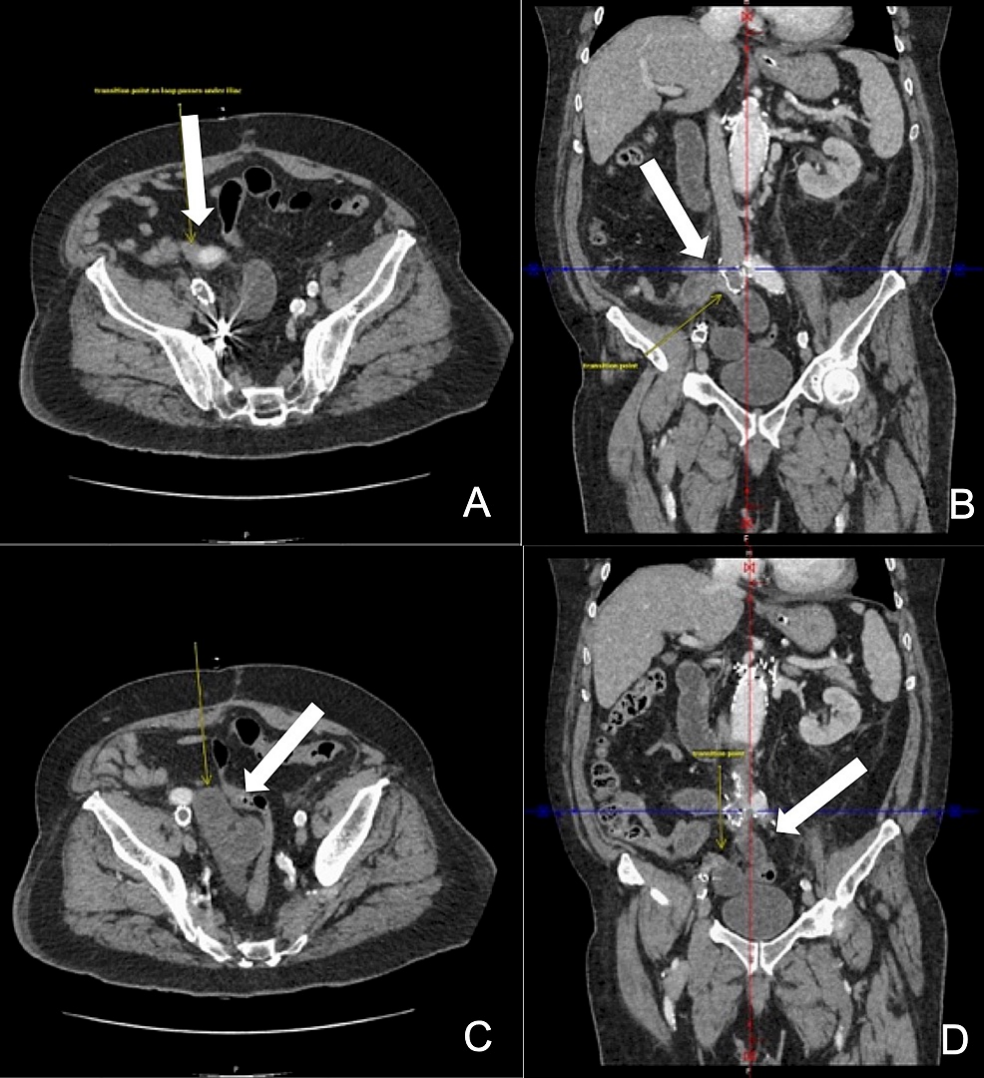
Computed tomography with intravenous contrast reveals a change in caliber as the bowel passes posterior to the right iliac graft limb (A,B). A dilated “C”- and “U”-shaped bowel loop can be visualized medial to the right iliac limb (C). Another transition point is seen in the more distal portion of the graft, consistent with closed-loop obstruction (D).

Computed tomography with intravenous contrast on postoperative day five revealed thickened and moderately dilated loops of the proximal small bowel with a more prominent C-shaped loop of the small bowel and two transition points in the right lower quadrant, medial and posterior to the right iliac segment of the aortobiiliac graft (Figure 1). The concern for nonviable or necrotic bowel prompted further investigation in the operating room.

Treatment

**Figure 2:**
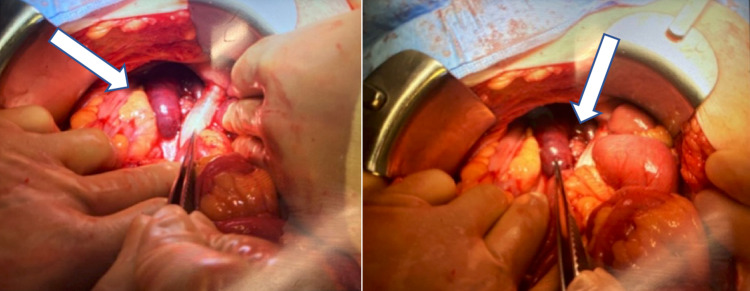
The jejunum courses posterior to the right iliac graft limb, and a 28-cm necrotic portion can be visualized medially.

The patient was taken emergently to the operating room for an exploratory laparotomy. Upon entering the abdominal cavity, extensive adhesions were encountered and lysed. While examining the small bowel, a jejunal loop was found fixed deep in the right hemi-pelvis. Upon further dissection, a 28-cm jejunal loop was entrapped behind the right iliac limb of the graft, causing a closed-loop jejunal obstruction and bowel necrosis (Figure 2). Extensive adhesions to the prosthetic graft were divided sharply. The jejunum was transected proximally and distally to the obstruction using a linear stapler device, and the necrotic bowel was resected. The patient was kept intubated, and the bowel was left in discontinuity with an open abdomen and a negative pressure therapy device.

He returned to the operating room the next day for a second-look laparotomy, and the remaining bowel appeared viable and well perfused. The jejunum was anastomosed, and the abdomen was closed. Over the next several days, the patient had return of bowel function. His diet was advanced, and he was discharged home on postoperative day eight. At two-week follow-up, the patient was ambulating, eating a regular diet, and performing routine daily activities.

## Discussion

This case highlights a rare intraoperative finding of jejunal strangulation posterior to a prosthetic aortobiiliac interposition graft. This closed-loop bowel obstruction occurred several days after elective coronary artery bypass grafting and aortic valve placement. When cardiopulmonary bypass is initiated during a coronary artery bypass graft, various hemodynamic changes take place. A patient’s mean arterial pressure decreases, and systemic vascular resistance decreases [5]. Carrel et al. analyzed 800 patients undergoing coronary artery bypass grafting and found a significant decrease in systemic vascular resistance in 21.8% of the patients [5]. With a lower mean arterial pressure and a potentially low-flow state, the bowel may have become edematous and precipitated strangulation of the jejunum posterior to the graft.

There is a higher incidence of bowel obstruction and overall reoperation rates with the open technique versus endovascular technique for aortic aneurysm repairs [4], likely related to the adhesive disease that developed during intraperitoneal manipulation. Adhesions of the bowel and mesentery to a prosthetic aortic graft have been reported [3-6] and may require surgical intervention if the bowel obstruction is refractory to conservative management with nasogastric suction. Bowel ischemia has also been reported after abdominal aortic aneurysm repair; however, this usually occurs in the acute postoperative setting and is most commonly related to graft thrombosis or ligation of the inferior mesenteric artery causing inadequate perfusion to the colon [4]. Prior to this report, there were no reported cases of a closed-loop obstruction or delayed bowel necrosis after an open abdominal aortic aneurysm repair.

In an unstable patient with peritonitis, emergent operative intervention is required [7]. In hemodynamically stable patients, computed tomography with intravenous contrast is a valuable diagnostic tool. There are particular imaging findings consistent with closed-loop obstructions. The “beak sign” is seen in 25% of patients and describes a beak-shaped configuration of the dilated bowel loops toward the obstruction [8]. A radial distribution of mesenteric vessels mimicking the spokes of a wheel (swirl) converging to the point of obstruction is another important predictor of bowel necrosis [8]. The most common diagnostic pattern of dilated bowel in closed-loop obstructions appears in a “U”- or “C”-shaped orientation; however, this can only be visualized with proper orientation in a single axial image [8].

## Conclusions

After an open transabdominal aortic aneurysm repair, extensive adhesive disease can form between the synthetic vascular graft and the surrounding visceral organs. When these patients undergo surgery with cardiopulmonary bypass, hemodynamic changes can occur, causing bowel edema, an increased susceptibility for bowel migration, and an entrapment posterior to the graft. A closed-loop obstruction by direct entrapment by the synthetic vascular graft should be considered.
